# Phase II Trial of Gemcitabine and Docetaxel with Bevacizumab in Soft Tissue Sarcoma

**DOI:** 10.1155/2015/532478

**Published:** 2015-05-14

**Authors:** Mark A. Dickson, David R. D'Adamo, Mary L. Keohan, Sandra P. D'Angelo, Richard D. Carvajal, Mrinal M. Gounder, Robert G. Maki, Li-Xuan Qin, Robert A. Lefkowitz, Olivia R. McKennon, Catherine M. Hirst, Gary K. Schwartz, William D. Tap

**Affiliations:** ^1^Department of Medicine, Memorial Sloan-Kettering Cancer Center and Weill Cornell Medical College, New York, NY, USA; ^2^Eisai, Inc., Woodcliff Lake, NJ, USA; ^3^Department of Medicine, Columbia University, New York, NY, USA; ^4^Departments of Medicine and Pediatrics, Mount Sinai School of Medicine, New York, NY, USA; ^5^Department of Biostatistics, Memorial Sloan-Kettering Cancer Center and Weill Cornell Medical College, New York, NY, USA; ^6^Department of Radiology, Memorial Sloan-Kettering Cancer Center and Weill Cornell Medical College, New York, NY, USA

## Abstract

Gemcitabine (G) and docetaxel (D) are commonly used to treat recurrent/metastatic soft tissue sarcoma. This study tested the hypothesis that outcomes would be improved by addition of bevacizumab (B). The initial design was randomized double-blind trial of G + D + B versus G + D + placebo. Due to slow accrual this was modified to single-arm open-label G + D + B. Eligible patients had diagnosis of leiomyosarcoma, pleomorphic undifferentiated sarcoma, pleomorphic liposarcoma, or angiosarcoma. Treatment was B 15 mg/kg on d1, G 900 mg/m^2^ on d1 and d8, and D 75 mg/m^2^ on d8, q21d. Primary endpoint was progression-free survival (PFS) at 6 months and would be met if ≥17 patients were progression-free at 6 m. Secondary endpoints are response rate, PFS at 3 m, overall survival, and toxicity. Of 44 patients enrolled, 35 were treated with GDB and evaluable for safety and efficacy. Median age was 55, 50% male, most ECOG 0. Toxicity is mostly myelosuppression with one deep vein thrombosis and one small bowel perforation possibly related to B. There were 17 partial responses (49%) by RECIST 1.1. Among 35 patients, the number who remained on study and progression-free was 24 at 3 m and 15 at 6 m. 9 withdrew prior to 6 m for reasons other than toxicity or progression. PFS at 6 m was 65% (95% CI: 51–85%). The primary endpoint of 6 m PFS was not met due to censoring of patients who withdrew. However PFS at 3 m (76%) was promising and response rate was higher than expected from G + D.

## 1. Introduction

The combination of gemcitabine and docetaxel for soft tissue sarcoma has been widely adopted over the last decade. This regimen was first examined in a phase 2 study in leiomyosarcoma which showed a high RECIST response rate of 53% [1]. A subsequent large retrospective analysis supported the activity of this regimen in multiple sarcoma subtypes but the reported response rate was lower at 18% [2]. A randomized prospective phase 2 study confirmed that gemcitabine and docetaxel combination was superior to gemcitabine alone in unselected histologies, with a response rate of 16% in the 73 patients treated with the combination [3]. Based on these data, gemcitabine and docetaxel combination has been widely adopted to treat recurrent sarcoma. In the published studies, most of the responses were in two histologies—leiomyosarcoma and undifferentiated pleomorphic sarcoma (UPS; previously known as malignant fibrous histiocytoma)—although responses were also seen in pleomorphic liposarcoma, rhabdomyosarcoma, and angiosarcoma [3].

This study attempted to improve on the standard gemcitabine and docetaxel regimen by adding the antiangiogenic drug bevacizumab. Bevacizumab is a humanized monoclonal antibody that binds vascular endothelial growth factor-A (VEGF-A). Bevacizumab enhances the effect of chemotherapy in multiple solid tumors and is FDA-approved for the treatment of lung, kidney, and colon cancer and glioblastoma.

Previous efforts at treating sarcoma with bevacizumab have met with limited success. Bevacizumab has some activity as a single agent in vascular sarcomas, for which it is a form of targeted therapy. The response rate is ~9% in angiosarcoma [4]. Bevacizumab has also been tested in combination with doxorubicin, which for many years has been the standard first-line regimen for recurrent sarcoma. In a phase II study, the response rate was modest at 12% but the study was closed due to excessive cardiac toxicity [5].

The current study tested the hypothesis that the addition of bevacizumab would enhance the activity of gemcitabine and docetaxel. The initial trial design was a double-blind, placebo-controlled, randomized trial of gemcitabine and docetaxel given with or without bevacizumab. Due to slow accrual, the trial was changed to a single-arm, open-label, nonrandomized study of gemcitabine, docetaxel, and bevacizumab, which we term here GDB.

## 2. Patient Eligibility

Eligible patients met the following criteria: histologically confirmed metastatic or locally recurrent leiomyosarcoma, undifferentiated pleomorphic sarcoma (UPS, formerly known as malignant fibrous histiocytoma, MFH), pleomorphic liposarcoma, pleomorphic rhabdomyosarcoma, or angiosarcoma, no more than 1 prior chemotherapy regimen for metastatic disease, measurable disease as defined by RECIST 1.1, ECOG performance status 0 or 1, age 18 to 75, and adequate hematologic, hepatic, and renal function. Patients with recent myocardial infarction, transient ischemic attack or stroke, congestive heart failure, brain metastases, uncontrolled hypertension, significant vascular disease, proteinuria, and recent abdominal fistula, perforation, or abscess were excluded. The protocol was approved by the Institutional Review Board of Memorial Sloan-Kettering Cancer Center and all patients provided written informed consent (Clinicaltrials.gov identifier NCT00887809).

## 3. Treatment Plan

Patients were treated with bevacizumab 15 mg/kg on day 1 of each 21-day cycle intravenously over 30 minutes. For cycles 1 through 6, patients were treated with gemcitabine 900 mg/m^2^ over 90 minutes on days 1 and 8 and docetaxel 75 mg/m^2^ over 60 minutes on day 8. Chemotherapy was started up to 60 minutes after the bevacizumab infusion. Treatment was followed by either 5 days of filgrastim or a single injection of pegfilgrastim. To improve tolerability for cycles 7 and beyond, gemcitabine was reduced to 800 mg/m^2^ over 30 minutes on days 1 and 8 and docetaxel was given at 35 mg/m^2^ over 30 minutes, also on days 1 and 8. Growth factors were not routinely used for patients receiving these doses beyond cycle 7.

## 4. Evaluation

A physical exam was done on day 1 and day 8 of the first two cycles and on day 1 of each subsequent cycle. Blood tests (complete blood count, serum chemistries, and liver enzymes) were performed prior to the start of every cycle and a complete blood count was repeated on day 8. Urinalysis was performed at every two cycles. Radiology assessments (CT or MRI) were performed every two cycles for the first 6 cycles, every 3 cycles for cycles 7–18, and every 4 cycles thereafter. Response was assessed by RECIST 1.1 [6].

## 5. Statistical Analysis

The initial study design involved a sample size of 72 patients, with 36 patients in the bevacizumab arm and 36 in the placebo arm. This study design would have allowed detection of a 75% improvement in median PFS in the bevacizumab arm, with type 1 and 2 error both set at 0.2. Due to slow accrual, the study was changed to a single-arm, nonrandomized trial of gemcitabine, docetaxel, and bevacizumab (GDB). For the revised study, a one-stage design was used with a sample size of 34. Patients previously enrolled in the randomized study were unblinded and only those who had received bevacizumab were included in the analysis. Accrual then continued until at least 34 evaluable patients had been treated with gemcitabine, docetaxel, and bevacizumab.

The primary endpoint for this study was progression-free survival (PFS) at 6 months. PFS includes both disease progression (as defined by RECIST 1.1) and death from any cause. Based on historical controls, a PFS of >60% at 6 months is considered promising, and a PFS of <40% is considered not promising [3, 7–9]. The study would be claimed to be positive if there were 17 or more who were progression-free at 6 months. This design has a type I error rate of 0.15 and a type II error rate of 0.09 based on an exact binomial test. Secondary endpoints included response rate by RECIST 1.1, PFS at 3 months, overall survival, and toxicity.

## 6. Results

**Figure 1:**
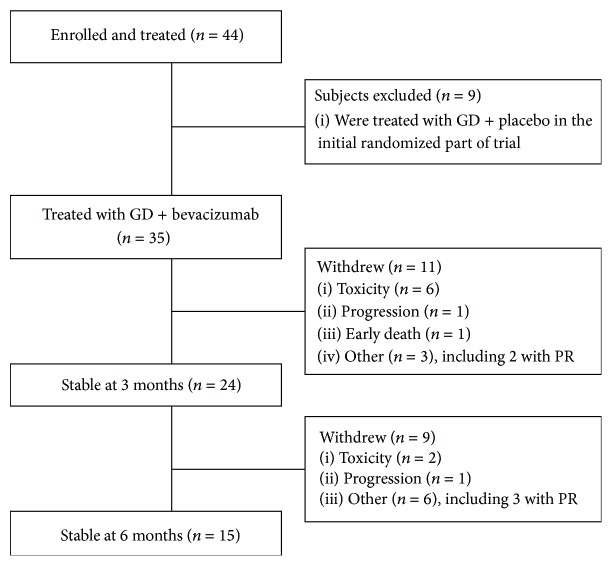
CONSORT diagram. Patients who received gemcitabine + docetaxel (GD) and placebo were excluded from the toxicity and efficacy analysis of GD + bevacizumab.

Between June 2009 and April 2010, 17 patients were enrolled in the randomized trial. At the time the study was changed to single-arm study of GDB, those 17 patients were unblinded. 9 had received placebo and were excluded from the safety and efficacy analysis. 8 had received bevacizumab. Between February 2011 and April 2012, an additional 27 patients were enrolled and treated with gemcitabine, docetaxel, and bevacizumab. Thus a total of 35 patients were treated with the combination and this population was used for the safety and efficacy analysis. The flow of these patients is shown in the CONSORT diagram in Figure 1.

The characteristics of all 44 patients enrolled are shown in Table 1. The median age was 55 (range 24–75) and 50% were male. Most patients had ECOG score of 0. The most common tumor types were leiomyosarcoma and UPS of the extremity or abdomen. Most patients (77%) had received no prior chemotherapy. The remainder had received one prior regimen of either doxorubicin or liposomal doxorubicin alone (5), doxorubicin with ifosfamide (4), doxorubicin + ifosfamide + dacarbazine (1), or sorafenib + dacarbazine (2—on a prior clinical trial).

### 6.1. Toxicity

The rates of adverse events are shown in Table 2. The most common adverse events were myelosuppression, consistent with prior reports of gemcitabine and docetaxel. One patient had a deep vein thrombosis that was likely related to metastatic sarcoma. One patient had a grade 2 small bowel perforation from diverticular disease that required a partial colectomy. This was considered possibly related to bevacizumab.

### 6.2. Efficacy

**Figure 2:**
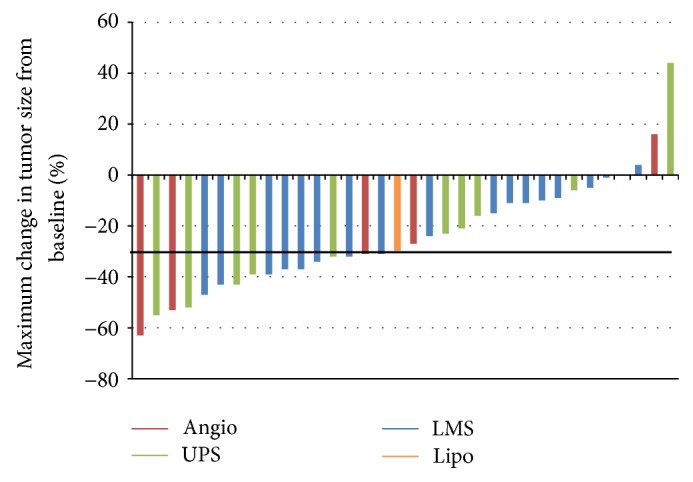
Waterfall plot showing tumor response in patients treated with gemcitabine + docetaxel + bevacizumab.

Of 35 patients, there were 17 partial responses, for an overall response rate of 49% (95% CI 31–66%). Responses were seen in all histologies: leiomyosarcoma (8/17 = 47%), undifferentiated pleomorphic sarcoma (5/11 = 45%), angiosarcoma (3/5 = 60%), and pleomorphic liposarcoma (1/1). Within the leiomyosarcoma group, 2/5 patients with uterine leiomyosarcoma had partial responses. In addition to the 17 partial responses, 9 patients had decrease in target lesions of at least 10% but not sufficient to meet criteria for PR. The best response by RECIST for each evaluable patient is shown in a waterfall plot in Figure 2.

Twenty four patients remained on treatment and progression-free at 3 months. The 11 patients who went off study before 3 months include 6 who stopped for toxicity, 3 who withdrew consent (see Figure 1 and Table 3), 1 who progressed, and 1 who died. Thus, when the patients who withdrew consent are censored, the PFS at 3 months is 76% (95% CI: 63–92%), based on the Kaplan-Meier method. (The outcomes of the 3 patients who withdrew consent before 3 months are described in Table 3. Two of the patients had PR by RECIST, and one was stable. Two underwent surgery and all 3 are without evidence of disease.) Between 3 and 6 months, an additional 2 patients stopped for toxicity, 6 withdrew consent (see Figure 1 and Table 3), and 1 progressed, resulting in 15 patients remaining on treatment and progression-free at 6 months. However the study required 17 patients to be progression-free at 6 months, so the primary endpoint was not reached.

**Figure 3:**
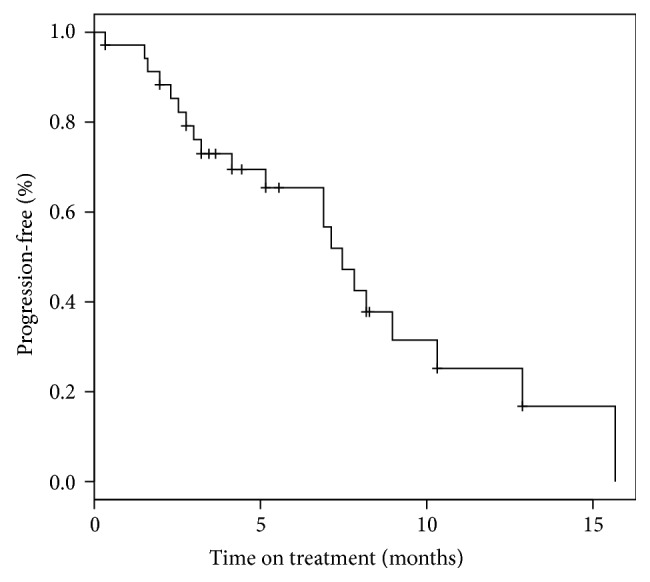
Progression-free survival.

**Figure 4:**
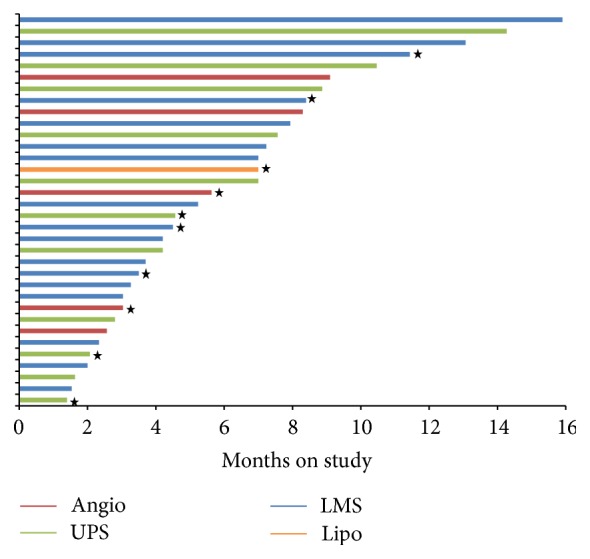
Duration on study for each patient treated with gemcitabine + docetaxel + bevacizumab. Patients marked by stars discontinued treatment on study because of good response and elected to have surgery.

A Kaplan-Meier curve of progression-free survival for all 35 patients is shown in Figure 3. The PFS at 6 months is 65% (95% CI: 51–85%); however this includes censoring for patients who withdrew. Note that 13 patients who had stable disease or partial response chose to stop protocol treatment for reasons other than progression or toxicity. Their outcomes are shown in Table 3. This group includes 9 patients who stopped treatment before reaching 6 months progression-free. The time on study for each evaluable patient is shown in Figure 4. Patients who were responding and elected to stop treatment so they could have surgery are indicated by stars. Patients who withdrew consent were censored for PFS analysis at the time of withdrawal. With this in mind, the median PFS for the 35 patients was 7.5 months (95% CI: 6.9-NR). The median overall survival was 2.4 years (95% CI: 1.9-NR).

## 7. Discussion

This study demonstrated the safety and potential efficacy of the combination of GDB for selected soft tissue sarcomas. The combination was generally tolerable and the most common toxicities were hematologic. This is consistent with the expected toxicity profile of gemcitabine and docetaxel. There did not appear to be a significant increase in toxicity due to the addition of bevacizumab, although conclusions drawn from a single-arm trial are limited. Several patients did withdraw from treatment due to toxicity. Although there was one episode of thrombosis and one episode of gastrointestinal perforation, overall the frequency of these events was not markedly higher than what has been reported in other solid tumors.

The objective RECIST response rate was 49% which compares favorably to what would be expected from gemcitabine and docetaxel alone, again taking into account the limitations of a single-arm phase 2 study. Only the initial phase 2 study in leiomyosarcoma reported a higher response rate (of 53% in 34 patients) [1]; however this was never replicated in subsequent studies. The pivotal randomized phase 2 study of gemcitabine and docetaxel versus gemcitabine alone showed a response rate of just 16% in 73 patients treated with the combination [3]. A second randomized phase 2 study in leiomyosarcoma showed a response rate of 24% for uterine and 5% for nonuterine LMS in a total of 90 patients [10].

A prior phase IB study of gemcitabine, docetaxel, and bevacizumab has been performed with a response rate of 31% in 36 patients [11]. In that study, however, the dose of bevacizumab was lower (5 mg/kg every 2 weeks, compared to 15 mg/kg every 3 weeks in this study) and the chemotherapy was given on an unusual schedule (gemcitabine 1500 mg/m^2^ and docetaxel 50 mg/m^2^ every two weeks). In contrast, the results of our study demonstrate that a higher dose of bevacizumab can be given with the standard doses of gemcitabine (days 1 and 8) and docetaxel (day 8) on an every-3-week schedule usually used in clinical practice.

After 6 cycles of treatment, we reduced the dose of docetaxel in all patients. It is our clinical practice to do this often in patients treated with conventional gemcitabine and docetaxel; however it has not been formally studied. The potential toxicity and efficacy of “split-dose” docetaxel warrant further study.

Radiographic responses occurred in all histological subtypes of sarcoma that were treated on this study. The results were particularly notable in visceral angiosarcoma where 4 of 5 patients had demonstrable tumor shrinkage and 3 met criteria for PR. This is a potentially encouraging result since the response rate of bevacizumab alone is low, and a recent randomized phase II study in angiosarcoma showed no benefit from the addition of bevacizumab to chemotherapy with paclitaxel [12]. Thus GDB may be a good option for angiosarcoma; however this hypothesis would have to be tested in a randomized trial.

This study also highlights the importance of choosing endpoints in phase II trials in sarcoma. Although this study did not meet its primary endpoint (17 evaluable patients progression-free at 6 months), this may have been due to the high withdrawal rate of patients for reasons other than progression. In particular, several patients who responded well elected to have surgery to resect residual metastatic disease, while others withdrew due to toxicity. Thus many patients became inevaluable for the primary endpoint of PFS at 6 months, confounding the results. A more standard benchmark for evaluating chemotherapy regimens in sarcoma is PFS at 3 months. By this standard, a 3-month PFS of at least 40% is considered promising in the second-line setting [9]. Although this was not the benchmark used in this study, the 3-month PFS of 76% would have compared favorably, except that most patients in this study were treated in the first-line setting and would therefore be expected to do better. In sum, this study suggests some favorable activity of GDB in certain sarcoma subtypes and also highlights the challenges of performing randomized trials in rare diseases and the importance of choosing consistent endpoints.

## Figures and Tables

**Table 1:** Patient characteristics.

Characteristic	All patients	GDB patients
Total	44	35

Male	22 (50%)	15 (43%)
Female	22 (50%)	20 (57%)
Age		
Median	54.5	54
Range	24–75	39–72
ECOG		
Median	0	0
Range	0-1	0-1
Tumor type		
Leiomyosarcoma	20 (45%)	18 (51%)
Uterine	5	5
Nonuterine	15	13
Undifferentiated pleomorphic sarcoma	17 (39%)	11 (31%)
Angiosarcoma (liver, bone, soft tissue)	6 (14%)	5 (14%)
Pleomorphic liposarcoma	1 (2%)	1 (3%)
Prior treatment		
Doxorubicin (or liposomal doxorubicin)	8	5
Ifosfamide	5	3
Dacarbazine	3	2
Sorafenib	2	1
None	34 (77%)	29 (83%)

**Table 2:** Adverse events occurring in more than 1 patient and all grade 3-4 adverse events.

Adverse event	Grade 2	Grade 3	Grade 4
Anemia	17	7	1
Neutropenia	7	7	6
Leukopenia	6	8	3
Hyperglycemia	12	3	1
Hypoalbuminemia	13	1	
Lymphopenia		11	2
Thrombocytopenia	1	4	5
Elevated ALT	9		1
Elevated AST	7	1	
Fatigue	1	5	
Hypophosphatemia	2	3	
Elevated alkaline phosphatase	2	2	
Hypokalemia		2	
Edema		2	
Diverticulitis		1	
Kidney stone		1	
Cellulitis		1	
Neck pain		1	
Pneumonia		1	
Mucositis	3		
Hemorrhage, nose	1		
Perforation, GI-small bowel NOS	1		
Thrombosis/thrombus/embolism			1

**Table 3:** Outcomes of patients who withdrew from treatment with GDB for reasons other than toxicity or progression (AWD = alive with disease; NED = no evidence of disease; DOD = died of disease).

	Time on treatment (months)	Best response (RECIST)	Maximal change of tumor (%)	Treatment after GDB	Pathology from surgery	Outcome
63 y male with recurrent UPS of thigh	1.4	PR	−39	Resection of local recurrence	Treatment effect but viable tumor	NED at 3.1 y
55 y female with recurrent UPS of thigh	2.1	SD	0	Limb perfusion and then surgery	30–40% necrosis and fibrosis	NED at 3.5 y
44 y female with angiosarcoma of vagina	3.0	PR	−63	Observation since biopsy showed no tumor	No tumor detected	NED at 1.3 y
55 y male with metastatic LMS	3.5	PR	−31	Resection of lung metastases	Necrosis and histiocytic infiltration (70%)	DOD at 1.4 y
50 y female with large pelvic (nonuterine) LMS	3.7	SD	−11	Evaluated for surgery but unresectable	N/A	AWD at 2.5 y
39 y male with metastatic LMS	4.2	SD	−15	Radio-frequency ablation of liver metastases	N/A	DOD at 2.4 y
69 y female with metastatic uterine LMS	4.5	SD	−24	Resection of pelvic metastases	Predominantly viable	AWD at 2.2 y
40 y male with metastatic UPS	4.6	PR	−32	Resection of lung metastases	Viable tumor	DOD at 1 y
52 y male with metastatic angiosarcoma	5.6	PR	−31	Resection of lung metastases	Viable but microscopic tumor	AWD at 11 m
56 y male with metastatic pleomorphic liposarcoma	7.0	SD	−30	Resection of solitary bone metastasis	>99% necrosis, rare viable cells	NED at 3.1 y
52 y male with metastatic LMS	8.4	PR	−32	Resection of lung metastases	95% treatment effect	AWD at 2.2 y
72 y female with metastatic UPS	8.9	PR	−55	Observation and then further chemotherapy	N/A	AWD at 2.6 y
57 y female with metastatic uterine LMS	11.4	PR	−43	Resection of lung metastases	30% treatment effect	DOD at 1.9 y
